# Experiences of Being Cared-For: The Perspective of an Expert-by-Experience in Mental Health

**DOI:** 10.3389/fpsyt.2022.824542

**Published:** 2022-03-10

**Authors:** Joanna Fox

**Affiliations:** School of Education and Social Care, Anglia Ruskin University, Cambridge, United Kingdom

**Keywords:** caregivers, carers, mental distress, autoethnography, expertise-by-experience

## Abstract

It is difficult to understand what it feels like for people with mental ill-health to be cared-for and supported by family members; this experience is often little-explored. Narratives about caring have been increasingly written alongside first-person accounts of recovery, however, there is a dearth of literature written to gain the perspective of being cared-for because of mental distress. Thus, using autoethnography, I present three critical incidents occurring at different points in my recovery to enable exploration of experiences of being cared-for. Firstly, a critical incident at the point of acute unwellness is introduced, secondly an incident during a consultation with a health professional is highlighted, and finally a moment of transition when embarking on an independent life with my husband-to-be is described. I use autoethnography to connect “the autobiographical and personal to the cultural, social, and political”. I consider how the identity of a carer is continually negotiated in a relationship with the service user in both the “private” and the “public” worlds during recovery. I reflect on how professionals can support both service users and carers in a triangle of care, by providing information and support, alongside promoting the development of independence and agency for the service user whilst in the caring relationship. Finally, I introduce a service model which promotes a family network approach to empower the service user, and highlight training programs on recovery that enable carers. I conclude by suggesting the potential of both approaches to support carers to promote the recovery of the service user.

## Introduction

It is difficult to understand what it feels like for people with mental ill-health to be cared-for and supported by family members; this experience is often little-explored. Narratives by caregivers have increasingly been written about their experiences of providing care to people with mental distress (1–3), in which they document frustrating and challenging, as well as joyous and hopeful moments of caring; however, it is clearly noted the tasks of caring that can impact negatively on both a person's physical and mental health (4). Of interest is a burgeoning body of first-person narratives written by experts-by-experience highlighting the nature of their recovery journey and experiences of mental ill-health (5), however there is a dearth of literature written to gain their perspective of being cared-for. There is furthermore a paucity of research to understand the perspective of being cared-for using experiential and autoethnographic methods. This article thus seeks to begin to bridge this gap in understanding this phenomenon.

In this article I reflect on my experiences of being cared-for by family members both when I was in the acute phases of psychosis and later when experiencing a long recovery. Using a process of reflection and the presentation of three critical incidents (6), I draw on autoethnography (7, 8) to explore my thoughts, feelings, and memories of being cared-for at different stages in my recovery journey. These reflective memories are contextualized in the wider social and cultural context (7, 8) and their relevance is highlighted.

### The Methodology: Telling Reflective Memories

Reflective practice is of key importance to both health and social care practitioners (9); it has a long tradition of being used in these professions as a method to develop both personal understanding of the lived experiences of service users and carers (10) and of innovation in practice (11). Autoethnography is a methodology that utilizes a process of reflective writing to provide an understanding of experiences in the wider social context (7, 8, 11). It has been increasingly used in health and social care research, education, and practice (11, 12). Autoethnography is thus employed in this article to explore my experiences of being cared-for, through a process of writing and reflection.

In this article I present three reflections, deliberately selected from critical points in my recovery journey because they evidence a step change in the experiences of being cared-for. These moments indicate a transition in the dyadic relationship between myself and my carers and mark a shift in my recovery journey. Alongside these three chosen moments of change, there is a concomitant reflection of the significance of this transition, thus allowing the connection of “the autobiographical and personal to the cultural, social, and political” (13). Throughout this process, autoethnographic writing (7) requires the researcher to pay careful attention to both the *epistemic* (claims to knowledge) and the *aesthetic* (practices of imaginative, creative, and artistic craft) characteristics of their texts as they seek to convey the meaning of their individual experiences and communicate their significance to the wider community of practice.

Critical incident reflections, as used in this article to capture my memories, are an approach often used in social work (6) and demonstrate how professional perspectives can provide new insights for service users, carers, practitioners themselves and their wider professional group. Although the content of the reflections is presented at a distance from my experiences, impacting on their potential accuracy, they still convey a very vivid description. Additionally, it could be argued that the accuracy of these stories may be blurred by the experiences of psychosis or could be unclear because of medication side effects; however, it is my contention that the value of first-person narratives is increasingly recognized and their role in highlighting the authentic experience of using services is highly regarded by service users, carers and many professionals (14). Moreover, as far back as the early 2000s, the importance of seeking the opinions and experiences of inpatients in mental health wards was recognized through the implementation of systematic user-focused monitoring (15). This highlighted the importance of listening to service users' opinions even when they were experiencing symptoms of mental ill-health or had blunted cognition because of the side effects of medication. In the next section, I provide three reflections on my experiences of being cared for, all of which occur at chosen points in my recovery.

### The Findings: Reflective Memories

*Reflection 1* took place at the beginning of first psychosis and extreme paranoia in 1990. I remember at the height of my terrors, when I was horrified at what I perceived to be happening around me, I was hearing voices in my head and had active symptoms of paranoia. At this moment I believed that I had superpowers and was going to be used by MI6 to stop terrorism in Northern Ireland. My parents had come up to Durham (in the north of England), where I was at university, knowing that something very distressing was happening to me, but not knowing what. I was sharing a room with my mother, as she stayed in a hotel in Durham whilst I was waiting for a psychiatrist's appointment. I was too terrified to stay in my room in college and became so scared that I crawled into bed with her, as I sought physical proximity to my mother to counteract the crisis I was experiencing. I now know the distress my mum experienced as she was unable to alleviate my crisis.

*Reflection 2* took place about 11 years later into my recovery in 2001. I remember one mental health appointment I went to. My mother came with me. I was working in London at that time and drove a 3-h round trip each day to my place of work and then back home. My mother drove me to the local hospital because I was tired of driving. She always drove me around locally. I self-managed my mental health and had control of choices about my medication. My psychiatrist suggested that it would be a good idea to come without my mother to the hospital. From my perspective, when my mother accompanied me to the hospital, it had nothing to do with her infantilising me or taking control of my life, rather it was a way of escaping the long driving. However, after that occasion, my mother never came with me again to the hospital. My psychiatrist challenged me in a quiet and professional way to become independent which impacted strongly on my sense of self as I strove to present myself as an adult. I couldn't have survived without the care of my mother, but she fostered a sense of dependency which to some extent infantilised me and removed my agency. As I reflect now, my mother was not able to “let go” in case I experienced a crisis or period of unwellness again.

*Reflection 3* took place at a time shortly before I was married in 2005. The final reflection is a turning point as I began an independent life and became responsible for myself, my home, and my own wellbeing. I had lived at home since leaving university at the age of 22. It was only when I met my husband-to-be and he expected me to be a grown adult and no longer a child, that I really grew up. I was entering an exciting and loving relationship and we had a new house together that needed a lot of work, and in the excitement of renovation I began to grow up. I was expected to do my fair share of household chores and to work full-time. I began to reduce the intimacy I had with my mother because her overwhelming care had become over-protective and intrusive. Maybe only now, as I reflect on my relationship with her at the time, do I realize the sense of fear and lack of control she felt in her caring role and the need to protect me when she felt she had failed me at the time of crisis in my first episode of psychosis. She longed to sustain a relationship of intimacy and support, although I rejected that bond. This relationship signified a status of dependency, weakness and vulnerability—all of which I rejected.

The relationship that I shared with my mother was central to my recovery journey. My mother constantly reinforced messages of hope and optimism, which are promoted in the recovery approach, to promote my wellbeing. Such concepts were under-developed at the time of my initial illness, but my mother played a pivotal role in my early recovery. My mother and I were co-dependent, and this relationship suited both her and my needs at different times in my recovery journey. Fostering a sense of dependency in my relationship with her, thus enabled her to protect me and to prevent any risk of harm, but at the same time to hinder any opportunity for growth. Additionally my mother was isolated and lacked the opportunity to learn from other caregivers. I felt uncomfortable with her going to a peer support group—I felt she was sharing my private information with other people, not understanding, as I do now, that she needed support and help to care in her own right. She thus had very little support in her own right, other than that of her husband, my father.

## Discussion

This section now seeks to connect “the autobiographical and personal to the cultural, social, and political” by enabling connections to be drawn between my own reflections on my relationship between my mother and me, and the wider research undertaken to consider the identity of family caregiver.

As shared in my reflections, the caring role is hard to define and understand, encompassing different tasks and different roles (16). Moreover the caregiver identity (17) is a socially constructed concept existing both in the public world of mental health services and professionals and in a private world between the carer and the cared-for. It has been socially constructed as a public identity (17) because the enactment of care in the community and the right to receive a carer's allowance and a carer's assessment, has led partners, parents, and siblings to be identified as caregivers. It has also been defined as existing in the private world between the caregiver and the cared-for as they individually negotiate their relationship of care, which is always changing and adapting. Thus, throughout the period of recovery, the caring/cared-for dyad is continually re-constructed in both the private world and the public world as the relationships change and develop, and are renegotiated between the cared for person and the caregiver. This is underlined in my reflections.

Moreover, my three reflections plot the changing relationship over time between my mother and me as we renegotiate our relationships and merge and separate our identities. Aldridge (2), a caregiver and mother of her son with undiagnosed bipolar disorder, highlight**s** the need for caregivers of people experiencing mental distress to balance both the support and care they provide against the need to respect the cared-for person's mental capacity and decision-making rights. She explores the difficulties of managing both care and control, while considering her own role of caring in a situation in which mental health services seemed unable to engage her son. It is often suggested by carers that this relationship should develop into a triad or a “triangle of care” (18) in which the service user, carer and professionals work together to support recovery of the service user. Aldridge (2) however experienced that service provision was missing, and professionals failed to provide appropriate care for her son. My reflections also revealed the importance of professionals including and supporting both me and my mother but reiterated the need to enable me to reassert my agency in treatment choices and appointments, disrupting the sense of co-dependency. Despite this, my mother emphasized a message of hope and optimism as suggested in the recovery model (19), as denoted in the CHIME model 1, and encouraged me to focus on my strengths (20). Such reinforcement can support people who experience mental distress to improve and sustain their sense of wellbeing, as my mother did in my case.

My reflections revealed the crucial role my mother played in my recovery, but also the co-dependency she fostered in our relationship. It is difficult for service providers to strike to correct position in supporting both the service users and the carer. As a population, caregivers often complain that they are under-supported and under-informed by services as they seek to care for their family members (2, 3). Although there may be conflict between the caregiver and the cared-for (21), most caregivers want to be positively involved in the lives of their family members. However, Henderson asks (18, p. 157) whether the caregiver and the cared-for experience any “shared interests and needs” or whether their needs are “incompatible and in conflict”. In recognition of these differing experiences, in England and Wales, service users have the right to displace their nearest relative (a legal position that allows them to make certain decisions in the enactment of the Mental Health Act) under section 29 of the Mental Health Act (22). Such an amendment to the law, recognizes that there may be conflict between some service users and carers, and they may not always share the same goals or objectives, suggesting the inadequacy of some relatives to occupy the role of nearest relative.

However, both caregivers and service users may have differing opinions of what constitutes best practice; for my mother it was hard for her to be told that I should be attending the mental health appointment by myself. Ryan (3) shares her experiences of caring for her son, diagnosed with Autistic Spectrum Disorder, describing both the effective as well as the unhelpful services they received from health and social care professionals. She documents how caregivers can feel excluded from professional support, which reduces their involvement in the life of the cared-for and prevents them from experiencing the respect and recognition as an ally. Furthermore, studies reveal that some caregivers experienced (23) powerlessness and lack of control as caregivers alongside a sense of failure from not preventing the admission, when service users entered the hospital. Additionally, they suffered feelings of isolation from the lack of partnership working as professionals took over the management of care. Moreover, one study (24) considers the importance of family caregivers participating in the lives of people who are currently expressing suicidal behavior and are residing in inpatient accommodation. It reiterates the needs for family members to be present and emotionally available for their relative, participating in their lives in such a way as to share everyday life; as well as to participate in joint activities that nurture sources for vitality and encourage thoughts about recovery and wellbeing. My reflections support the contention that professionals should use their professional judgement to recognize the needs of service users at different times in their care, and to use their professional knowledge to enable the caregiver to provide the most effective possible support.

Taking these experiences into account, practice models, such as the Triangle of Care (18), encourage mental health service professionals to involve caregivers in supporting service users. The Triangle of Care is a model which attempts to establish therapeutic alliance between the service user, professional and caregiver. Partnership models are important for different members of the care team at different points in the recovery journey (1); however, these need to recognize when dependency is preventing the independent growth and recovery of the service user. Moreover, internationally, the Open Dialogue programme (25) implemented in Finnish Lapland uses a family-centered approach that focuses on recognizing the significance of all members of the service user's network in their assessment and treatment. This involves regular meetings of all stakeholders in the group with treatment lasting for up to 2 years. It is of increasing influence in the UK (26) and elsewhere—although, with its focus on the user's right to confidentiality and autonomy, practice in the UK would require significant change to implement fully this model.

Finally, caregivers need information and advice to enable them to care effectively, although they have historically received little training or support in their caring role (27). However, recently the implementation of caregivers' education programmes is now recommended in UK government guidance (28), and caregivers are given a right to support in accordance with the Care Act (29). Furthermore, training opportunities have become increasingly available for caregivers of people with specific mental health conditions, such as personality disorder (30, 31) or schizophrenia (32). Despite this, access to forms of peer support and training are often denied to many caregivers by the cared-for person's fears (33). This underlines the need for accessible and sensitive training to support caregivers to care effectively (34). Research I have undertaken (33, 34) has led to the development of a training programme on the recovery approach for family caregivers, co-produced by different stakeholders and co-delivered by myself, in my identity as a service user, and with a caregiver. This programme allows me to share my experiences of mental ill-health to enable carers to understand mental distress and the enablers and barriers to recovery (34). This programme reinforces the intention of this paper to enable carers and service users to reflect on and renegotiate their relationship in the caring dyad.

## Limitations

This article attempts to enable carers to understand better the potential enablers and barriers to an effective relationship with their family member in the process of recovery. However, a potential limitation, is that the autoethnographic methodology itself allows the writer of the reflections to be the primary analyser of the perceptions. This leads to the potential of bias in the process. However, autoethnography emphasizes the primacy of the individual at the center of both the narrative and the analysis and acknowledges the importance of the *self* interacting with the *social context* (7, 8, 13). This thus reinforces the value of the person making sense of their individual experiences through a process of reflexivity and undertaking meaning-making through the connection of the personal to the political (13). It is this individual experience that is thus validated through the process of reflection, recognized as meaningful, and acknowledged as having an important role to play in the construction of knowledge (11).

## Conclusion

This article has highlighted the experiences of being cared-for from the perspective of an expert-by-experience, using autoethnography (7, 8, 13). I have presented three critical incidents during my recovery journey of my experiences of being cared for, and through wider analysis of these occurrences sought to connect “the autobiographical and personal to the cultural, social, and political” (13). This reflection has led to consideration of the important role that professionals can play in supporting both the service user and the caregiver to renegotiate their relationships in their caring dyad, to foster independence but to build alliances with carers in a triangle of care (18). Such demands highlight the need for professionals to use their professional judgement to support recovery as they work with both the service user and the carer.

Furthermore, the importance of involving caregivers in service users' lives is increasingly recognized (1–3), which has led to the development of effective service partnership models (25, 26) to increase the influence of family members in the care of the person with mental distress. Training programmes which share authentic experiences of recovery, co-produced and co-delivered by service users and carers also play an important part in providing information and support to caregivers (33, 34). This article thus concludes the need for caregivers to hear more of service user's experiences of recovery and of being cared-for. Thus, this article seeks to contribute to this process and to support caregivers' effective involvement in the care of their family member.

## Data Availability Statement

The original contributions presented in the study are included in the article/supplementary material, further inquiries can be directed to the corresponding author.

## Author Contributions

JF wrote and developed this article.

## Conflict of Interest

The author declares that the research was conducted in the absence of any commercial or financial relationships that could be construed as a potential conflict of interest.

## Publisher's Note

All claims expressed in this article are solely those of the authors and do not necessarily represent those of their affiliated organizations, or those of the publisher, the editors and the reviewers. Any product that may be evaluated in this article, or claim that may be made by its manufacturer, is not guaranteed or endorsed by the publisher.
